# Plasma cytokine profiles associated with *rhodesiense* sleeping sickness and *falciparum* malaria co-infection in North Eastern Uganda

**DOI:** 10.1186/s13223-019-0377-7

**Published:** 2019-10-30

**Authors:** Julius Nsubuga, Charles Drago Kato, Ann Nanteza, Enock Matovu, Vincent Pius Alibu

**Affiliations:** 1 College of Veterinary Medicine, Animal Resources & Bio-security, Makerere University, Kampala, Uganda; 2 College of Natural Sciences, Makerere University, Kampala, Uganda

**Keywords:** HAT, Malaria, Co-infection, Mono-infection, Cytokine, IFN-γ, TNF-α, IL-6, IL-10, TGF-β

## Abstract

**Background:**

Immunological Human African Trypanosomiasis (HAT) studies often exclude malaria, although both infections overlap in specific endemic areas. During this co-infection, it is not known whether this parasitic interaction induces synergistic or antagonistic cytokine response among humans. This study determined prevalence of *Plasmodium falciparum* malaria among *Trypanosoma brucei rhodesiense* HAT and plasma cytokine profile levels associated with HAT and/or malaria infections.

**Methods:**

Participants were recruited at Lwala hospital in north eastern Uganda: healthy controls (30), malaria (28), HAT (17), HAT and malaria (15) diagnosed by microscopy and PCR was carried out for parasite species identification. Plasma cytokine levels of Interferon-gamma (IFN-γ), Tumour Necrosis Factor-alpha (TNF-α), Interleukin (IL)-6, IL-10 and Transforming Growth Factor-beta (TGF-β) were measured by sandwich Enzyme-Linked Immuno Sorbent Assay and data statistically analysed using Graphpad Prism 6.0.

**Results:**

The prevalence of *P. falciparum* malaria among *T. rhodesiense* HAT cases was high (46.8%). Malaria and/or HAT cases presented significant higher plasma cytokine levels of IFN-γ, TNF-α, IL-6, IL-10 and TGF-β than healthy controls (P < 0.05). Levels of IFN-γ, IL-6 and IL-10 were significantly elevated in HAT over malaria (P < 0.05) but no significant difference in TNF-α and TGF-β between HAT and malaria (P > 0.05). Co-infection expressed significantly higher plasma IFN-γ, IL-6, and IL-10 levels than malaria (P < 0.05) but no significant difference with HAT mono-infection (P > 0.05). The TNF-α level was significantly elevated in co-infection over HAT or malaria mono-infections (P < 0.05) unlike TGF-β level. Significant positive correlations were identified between IFN-γ verses TNF-α and IL-6 verses IL-10 in co-infection (Spearman’s P < 0.05).

**Conclusions:**

The *T. b. rhodesiense* significantly induced the cytokine response more than *P. falciparum* infections. Co-infection led to synergistic stimulation of pro-inflammatory (IFN-γ, TNF-α), and anti-inflammatory (IL-6, and IL-10) cytokine responses relative to malaria mono-infection. Level of TNF-α partially indicates the effect induced by *T. b. rhodesiense* and *P. falciparum* mono-infections or a synergistic interaction of co-infections which may have adverse effects on pathogenesis, prognosis and resolution of the infections.

*Trial registration* VCD-IRC/021, 26/08/2011; HS 1089, 16/01/2012

## Background

Human African Trypanosomiasis (HAT) and/or malaria are two protozoan parasitic vector borne diseases of public health concern in endemic areas of Africa. In Uganda, malaria is prevalent throughout the country whereas HAT is endemic in specific foci. Nevertheless, malaria coincides with HAT in specific overlapping areas. Therefore plasmodium and trypanosome infection may exist within a host concurrently [1, 2]. This is because tsetse flies and mosquitoes that transmit HAT and malaria parasites respectively, share these specific co-endemic habitats [3]. This co-infection is of epidemiological and immunological importance, although most studies focus on single-pathogen infections. Studies conducted in East Africa showed malaria prevalence among HAT cases of 28.9% in eastern Uganda [2], 2.9% in Uganda, 79.7% in Tanzania [1], and 100% in Kenya [3]. Also, HAT cases present clinical symptoms similar to those of malaria [4]. This makes diagnosis and management of both diseases difficult in overlapping areas. Nevertheless, HAT clinical symptoms and fatality are not significantly affected by malaria [1, 2].

At immunological level, quite different immune cytokine responses are induced by different parasites. Studies in animal and human co-infections suggest significant variation in the immune cytokine response to occur in reaction to pathogen–pathogen interactions. This is essential in the pathogenesis of infections (pathogen–host interactions) and their interactions (pathogen–pathogen interactions) in the host. During co-infections, the parasites may exhibit synergistic, antagonistic, or competitive interactions with the immune system of host [5, 6]. The pro-inflammatory or anti-inflammatory cytokines secreted may cause cross-immunity or immune-suppression leading to partial host protection and regulation of infections. When these parasitic interactions induce immunosuppression, it enhances parasite survival and pathogenicity in the host [5]. Cytokine modulation is thought to be associated with the degree of clinical manifestation, parasitemia, pathogenesis, disease severity and survival among HAT [7–9] or malaria cases [10–12]. The pro-inflammatory cytokines (like IFN-γ) are important in parasite control during early stage infection by activation of TNF-α and nitric oxide (NO) secretion from macrophages, and then a switch to anti-inflammatory cytokines (IL-10, IL-4) with protective immunity crucial for survival of host or parasite during late and chronic stages of trypanosomiasis [13, 14]. Similarly pro-inflammatory cytokines are essential in the resolution of parasitemia, clinical manifestation and pathogenesis, especially during the early stages of *P. falciparum* infection [11, 12, 15]. Anti-inflammatory cytokines like TGF-β are produced to regulate pro-inflammatory responses and secretion [16]. Also identified for its anti-inflammatory protective role in autoimmune conditions [17], IL-10 act as an immunoregulator neutralizing the effects of inflammatory responses associated with immunopathology and severe forms of *P. falciparum* infection [10, 18]. In animal models, IFN-γ level was described to be more elevated in *T. brucei* than *Plasmodium berghei* infected mice. Co-infected mice expressed elevated IFN-γ and TNF-α levels over *P. berghei* or *T. brucei* mono-infected group suggesting active response against secondary infection. Although IFN-γ in co-infected mice was more less than in *T. brucei* mono-infected group. The induction towards pro-inflammatory response (TNF-α, IFN-γ and NO) by *T. brucei* could account for plasmodium hepatic impairment in mice [19]. However, anti-inflammatory IL-10 plasma level was significantly lower in *T. b. gambiense* and *P. falciparum* co-infection than healthy controls. Consequently, IL-10 plasma level of HAT was similar irrespective of *P. falciparum* infection [20].

The plasmodium and trypanosome infections have been extensively described separately with potential to induce cytokine production in the host. Despite epidemiological HAT and malaria co-infection studies, immunological cytokine response to *T. b. rhodesiense* and *P. falciparum* co-infection, and its relative comparison to mono-infection in naturally infected cases are not or poorly explored. Immunological HAT studies have always excluded malaria although both infections overlap in specific co-endemic areas of tropical Africa [8, 10]. It is not known whether this parasitic interaction induces synergistic or antagonistic cytokine response among co-infected humans relative to either mono-infection. The study determined prevalence of *P. falciparum* malaria among *T. b. rhodesiense* HAT cases and plasma cytokine profile levels associated with parasitological acute sleeping sickness and/or malaria cases from north eastern Uganda. The results will provide insights that can be manipulated in future to aid clinicians, diagnostic approaches, vector or disease control policy team on how to handle HAT and malaria cases. The comparison of cytokine concentration examined how *T. b. rhodesiense* HAT and *P. falciparum* malaria co-infection modify the immunological cytokine response induced by the two parasites relative to mono-infections. This will contribute to the understanding of the immunological response of this co-infection and management of cases, emphasising the significance of immune-mediated interactions in poly-parasitism among people.

## Materials and methods

### Study area

**Fig. 1:**
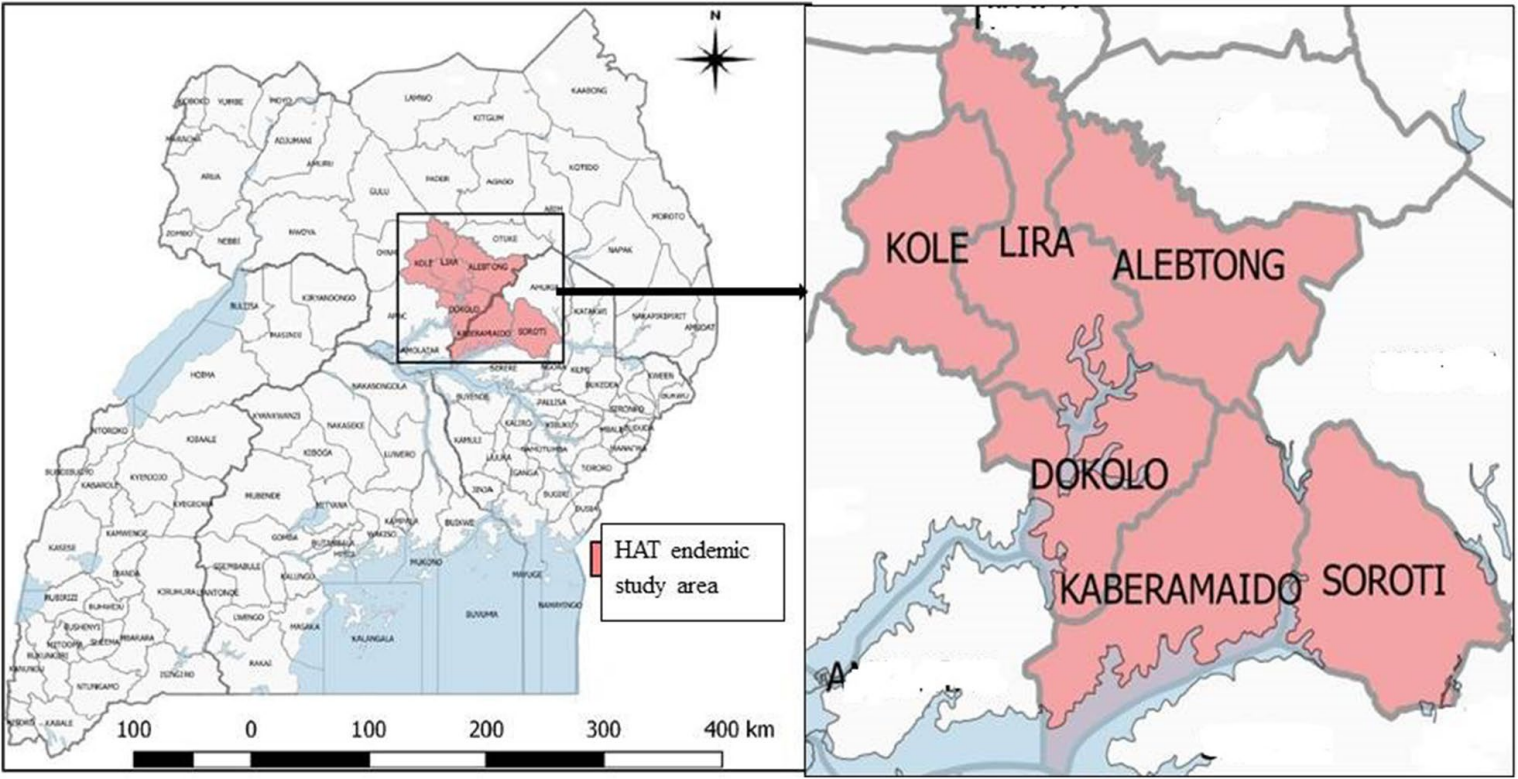
Map of Uganda showing the six districts with Human African Trypanosomiasis and malaria cases reporting at Lwala hospital. Map of Uganda was sourced from Uganda bureau of statistics [27] and modified. Map of the study area was drawn using QGIS

Participants were recruited from north eastern Uganda at Lwala hospital, a sleeping sickness referral center in Kaberamaido district, providing health services especially to HAT cases. Since 2004, this area has been affected by *T. b. rhodesiense* HAT which extended from the historical foci in the eastern part of the country [21]. Currently, HAT has been identified to be prevalent from a large endemic area of Dokolo, Kaberamaido, Soroti, Lira, Alebtong, and Kole districts (Fig. 1). Within this area, an approximation of 7.9 million people are at risk of acquiring *T. b. rhodesiense* sleeping sickness [22]. The highest occurrence of the disease has been noted in Dokolo and Kaberamaido with 60.9% of *T. b. rhodesiense* infected cattle as human reservoirs [23]. Malaria is also prevalent in north eastern Uganda that coincides with HAT [2].

### Study design and sample collection

This was a case control study to determine the prevalence of *P. falciparum* malaria among *T. b. rhodesiense* HAT cases and associated plasma cytokine levels of HAT and/or malaria cases from the region reporting to Lwala hospital, aged 6 years and above between October/2013 and May/2014. Patients report to hospital with common complaints of; fever (temperature > 37 °C), general body weakness, headache, joint pain, tremor, loss of appetite and sweating. It is a routine practice at Lwala hospital to microscopically check for malaria and HAT parasites. After patients had given detailed clinical descriptions to the medical physician, participants were subjected to routine laboratory diagnostic procedures at the hospital required to guide treatment. Microscopic examination of wet and thick blood films from finger prick blood stained with 10% Giemsa or Field stain for plasmodium and trypanosomes [4, 24], or haematocrit centrifugation technique (HCT) for trypanosomes [25] were performed. After detection of parasites in participants, blood (5 ml) by venepuncture using EDTA vacutainer tubes (BD-Plymouth, UK) was collected after consent before treatment for HAT and/or malaria. If blood smear was positive for trypanosomes or patient presented with HAT signs (convulsions, tremors, psychotic behaviour and sleep disorders), lumber puncture was performed to obtain CSF (3 ml) following WHO sleeping sickness staging guidelines [22]. White blood cell (WBC) count in CSF was performed by Neubauer Haemocytometer. Analysis of CSF for trypanosomes was executed by the modified single centrifugation method [26]. The presence of trypanosomes in blood but absent in CSF or WBC count < 5 cells/mm^3^ was categorised as early stage HAT while late stage infection was confirmed by the presence of trypanosomes in the CSF and/or WBC count > 5 cells/mm^3^.

Following this diagnosis, patients were recommended for the appropriate treatment and monitoring by the clinician. For HAT, this involved administering Suramin and Melarsoprol for early and late stage HAT respectively [22]. Malaria patients were treated with Artemisinin-based combination therapy (ACT) [27]. Healthy controls were mobilised from their homes within the study area by the principal investigator and the field team to the hospital with prior knowledge about the study purpose. The blood collected was subjected to the routine laboratory diagnosis at the hospital. The collected blood (5 ml) from cases and controls was centrifuged for 10 min at 3000 rpm using Eppendorf centrifuge (5424R, Germany) to obtain plasma and packed cell volume (PCV) of blood. The plasma (2 ml) and PCV (2 ml) were aliquoted and stored in liquid nitrogen. The participant samples were transported to Afrique One laboratory, College of Veterinary Medicine, Animal Resources and Biosecurity (COVAB), Makerere University for SRA or MSP-2 PCR and cytokine analysis. The sample size was calculated using G*Power 3.0 software giving a total of 90 participants considered for the study. The minimum number per group (HAT-malaria, HAT, malaria and healthy controls) for cytokine analysis was 15 participants (Power = 80% and P = 0.05).

### Inclusion and exclusion criteria

All HAT and/or malaria cases and healthy control individuals diagnosed from the region reporting at Lwala hospital were included, after a written informed consent in their local language (Kumam) from adults or guardians in case of minors (6 < 18 years) in addition to their assent. A case was an individual with positive blood smear of trypanosome and/or plasmodium parasites in blood, or presence of trypanosomes in CSF. For parasite species identification, SRA (*T. b. rhodesiense*) and/or MSP-2 (*P. falciparum*) nested PCR was carried out. A healthy control was an individual without any detectable parasite on blood smear or other infections and SRA or MSP-2 PCR negative. Individuals diagnosed with infections of helminths (microscopic examination of filtered urine for the presence of eggs), amebiasis (detection of cysts in stool), typhoid (Widal method), tuberculosis (sputum microscopy), human immunodeficiency virus (HIV) (rapid diagnostic test strip) and Urinary tract infections (UTI) (urine test strip compared to coloured scale) [4] were excluded. Children below 6 years were excluded from the study due to their developing and differences in capability of immune system [28]. Previously HAT and/or malaria treated individuals before a lapse of 1 month to eliminate effect of slow acting drugs [29] and those who did not consent to the study were excluded from study.

### Amplification of *Trypanosoma brucei rhodesiense* serum resistance associated gene

The extraction of DNA from blood PCV was performed using QIAGEN DNeasy Blood & Tissue Kit (Qiagen^®^, USA) according to manufacturer’s instructions. The eluted DNA was stored at − 20 °C for PCR analysis. Nested SRA PCR was used to detect *T. b. rhodesiense* using sequence primers (Microsynth, Switzerland) on test samples, positive control (Genomic DNA extracted from previously characterised strain of *T. b. rhodesiense* from Tororo endemic area) or negative control (PCR water, Bioline, USA) with conditions as described by Maina et al. [30] and products visualized under UV on 2% gel stained with 0.5 μg/ml ethidium bromide (Sigma, USA).

### Amplification of *Plasmodium falciparum* merozoite surface protein-2 gene

Nested MSP-2 PCR was used to detect the presence of *P. falciparum* using sequence specific primers ((Biolegio, Netherlands) on test samples, positive control (3D7 or HB3 Genomic DNA extracted from cloned strains, Invitrogen, USA) or negative controls (PCR water, Bioline, USA) with conditions as described by Dokomajilar et al. [31] and products visualized under UV on 2% gel stained with 0.5 μg/ml ethidium bromide (Sigma, USA).

### Plasma cytokine assays

The selected cytokines (IFN-γ, TNF-α, IL-6, IL-10 and TGF-β) have been associated with the pathogenesis, disease severity and survival in either HAT [7–9] or malaria [11, 12, 15]. Plasma concentrations of cytokines (IFN-γ, TNF-α, IL-6, IL-10, and TGF-β) were assayed using solid phase sandwich ELISA kit (BD OptEIA™, San Diego, USA) as described previously [8, 32]. Briefly, 96 well microplates (nunc™, Denmark) were coated with 100 μl per well of capture antibody diluted in coating buffer (1× phosphate-buffered saline, PBS) and incubated over night at 4 °C (Electrocool LG, South Korea). Microplates were aspirated and washed 3 times with 300 µl of wash buffer. Microplates were then blocked with 200 µl of assay diluent containing 10% fetal bovine serum albumin (Biochrom^AG^, Berlin) and incubated for 1 h at room temperature (RT). After washing microplates 3 times with wash buffer, 200 µl assay diluent was added, followed by 100 µl plasma sample, 100 µl standards (OptEIA, Belgium), serially diluted in 100 µl assay diluent and 100 µl controls and incubated for 2 h at RT. After washing microplates 5 times, 100 µl working antibody detector (biotinylated detection antibody + streptavidin–horseradish peroxidase) was added and incubated for 1 h at RT. After 7 washes, 100 µl substrate solution (tetramethylbenzidine, TMB, BD Biosciences, Belgium) was added and incubated in the dark for 30 min for colour development, after which 50 µl stop solution (2 M H_2_SO_4_) was added and the plate read at 450 nm using a microplate reader (Biotek, UK). All assays were done in triplicate wells on the same plate and readings averaged. For TGF-β, the plasma sample was centrifuged for 2 h at 1800×*g* to release the latent TGF-β1 into plasma and then activated through acidification with 1 M HCl at 4 °C for 60 min and neutralised with 1 M NaOH. Cytokine concentrations of test samples were extrapolated from the optical density (OD)-concentration standard curve obtained from serial dilution of recombinant cytokine standards using GraphPad Prism 6.0 statistical package.

### Data analysis and management

All numerical variables were summarized using mean and standard deviation of mean. All comparisons of categorical variables and cytokine data were analyzed using Graphpad Prism 6.0 statistical packages. Comparison of categorical variables was performed using Chi-square test at P < 0.05 significance. Deviation from normality was tested using D’Agostino and Pearson omnibus normality test of plasma cytokine data. Cytokine data was presented as medians since it did not present a normal distribution. Comparison of cytokine levels between the different groups of participants was done using nonparametric tests; Mann–Whitney U and Kruskal–Wallis tests followed by Dunn’s multiple comparisons test at a significant level (P < 0.05, two tailed). Correlation analysis between cytokines was performed using bivariate non-parametric Spearman’s correlation rank test at a significant level (P < 0.05, two tailed).

## Results

### Demographic and baseline characteristics of participants

A total of 60 cases and 30 healthy controls recruited at Lwala hospital were enrolled for the study after hospital microscopy diagnosis, HCT and PCR parasite species identification. The ratio of male (48) to female (42) was approximately 1:1 with an average age of 28.8 ± 14.1 years. Participants 18 years and above were significantly more infected than those of six and below 18 years (P < 0.05). There was no significant association between sex and infection status (P > 0.05). There were 14 early and 18 late stage HAT cases in this study with no significant difference (P > 0.05) (Chi-square, P < 0.05). Among the participants; malaria mono-infected cases were 31.1% (28/90), 18.9% (17/90) were HAT mono-infected, while 16.7% (15/90) had HAT and malaria co-infection and 33.3% (30/90) were healthy controls. Among the 32 HAT cases, 15 were positive for *P. falciparum* malaria. Therefore, the prevalence of malaria among HAT cases was 46.8% (15/32) (Table 1).

**Table 1:** Demographic and baseline characteristics of participants

Characteristic	Participant status					P value
	Malaria	HAT	HAT + malaria	Healthy controls	Total n (%)	
Subjects n (%)	28 (31.1)	17 (18.9)	15 (16.7)	30 (33.3)	90 (100)	
Average age	25.1 ± 11.1	26.5 ± 13.4	22.3 ± 14.5	36.4 ± 14.4	NA	
Sex						
Male	15	9	10	14	48 (53.3)	> 0.05
Female	13	8	5	16	42 (46.7)	
Age group (years)						
6 < 18	4	5	5	1	15 (16.7)	< 0.05^a^
≥ 18	24	12	10	29	75 (83.3)	
HAT stage						
Early	NA	7	7	NA	14 (47.8)	> 0.05
Late	NA	10	8	NA	18 (56.2)	

*HAT* Human African Trypanosomiasis, *NA* not applicable

^a^Significantly higher in participants ≥ 18 years, 6 < 18; young adults, ≥ 18; adults

### Plasma cytokine levels in HAT and/or malaria infections relative to healthy controls

**Fig. 2:**
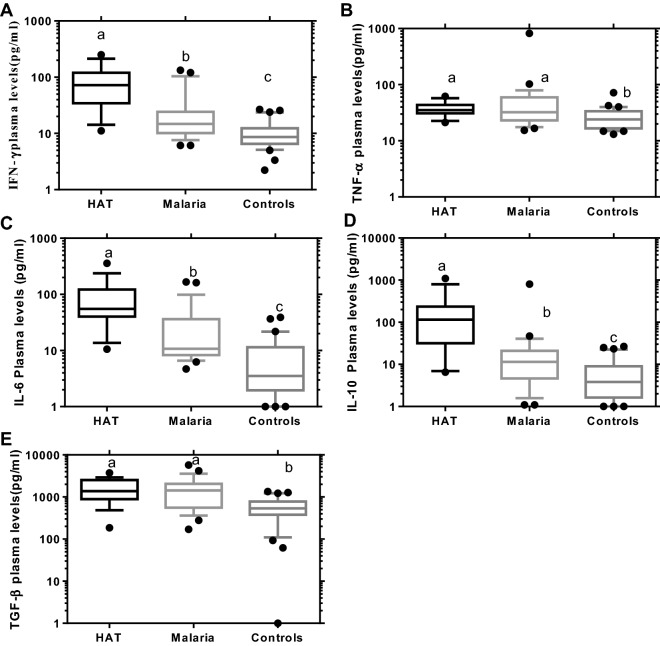
Plasma cytokine levels of HAT or malaria mono-infections compared to healthy controls. Participants of HAT (n = 17) or malaria (n = 28) mono-infections and healthy controls (n = 30) were involved. Boxes indicate median and interquartile range, whiskers are defined as 10th–90th percentiles. Dots define outliers. Letters (a > b > c) indicate level of significance between participant groups (Kruskal–Wallis, Dunn’s multiple comparisons tests, P < 0.05)

**Fig. 3:**
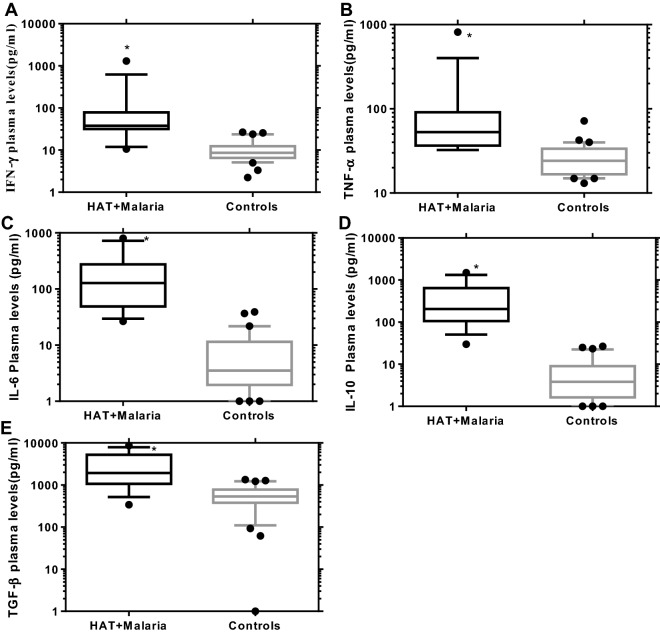
Plasma cytokine levels of HAT and malaria co-infection compared to healthy controls. Participants of HAT and malaria co-infection (n = 15) and healthy controls (n = 30). Boxes indicate median and interquartile range, whiskers are defined as 10th–90th percentiles. Dots define outliers. Asterisk (*) indicate significant elevation in co-infected cases over healthy controls (Mann–Whitney U test, P < 0.05)

The assay was carried out for five plasma cytokines (IFN-γ, TNF-α, IL-6, IL-10, and TGF-β) in HAT and/or malaria cases and healthy controls. The detection limits for the cytokine assays (IFN-γ, TNF-α, IL-6, IL-10, and TGF-β) were 8.3, 9.1, 3.6, 4.2, and 522.2 pg/ml respectively, calculated according to Armbruster and Pry [33]. The plasma levels of IFN-γ, IL-6 and IL-10 were higher in HAT (72.23, 55.04 and 115.5 pg/ml) or malaria cases (14.72, 10.74, and 11.44 pg/ml) respectively than healthy controls (8.612, 3.513, and 3.812 pg/ml) (HAT significance: P < 0.0001, P < 0.0001, P < 0.0001 or malaria significance: P = 0.0169, P = 0.0015, and P = 0.0288) respectively. These cytokines were also significantly elevated in HAT over malaria cases (P = 0.0027; P = 0.0060; and P = 0.0057, Fig. 2a, c, d) respectively. Similarly, plasma TNF-α and TGF-β level of HAT (35.15 and 1379 pg/ml) or malaria (32.22 and 1434 pg/ml), was significantly elevated over healthy controls (24.11 and 534.7 pg/ml), (HAT significance: P = 0.0052 and P = 0.0003 or malaria significance: P = 0.0103 and P = 0.0009) respectively. However, no significant difference in the level of these cytokines (TNF-α and TGF-β) between HAT and malaria cases was noted (P > 0.9999), (Fig. 2b, e respectively, Kruskal–Wallis, Dunn’s multiple comparisons test, P < 0.05). The HAT and malaria co-infection expressed significantly higher median plasma cytokine level of IFN-γ, TNF-α, IL-6, IL-10, and TGF-β (37.78, 52.72, 127.5, 205.3, and 1922 pg/ml) respectively than healthy controls (P < 0.0001, Fig. 3) (Mann–Whitney U test, P < 0.05).

### Plasma cytokine level in HAT and malaria co-infection relative to mono-infections

**Fig. 4:**
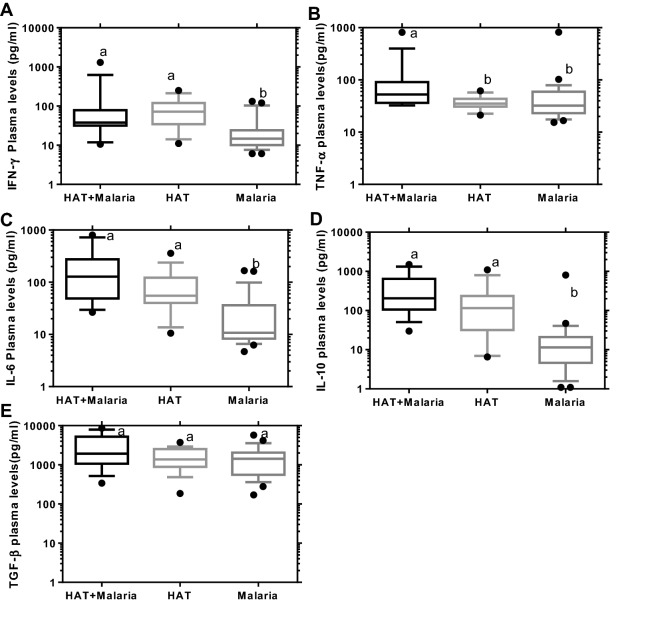
Plasma cytokine levels of HAT and malaria co-infection compared to mono-infections. Participants of HAT and malaria co-infection (n = 15), HAT (n = 17) or malaria (n = 28) mono-infections. Boxes indicate median and interquartile range, whiskers are defined as 10th–90th percentiles. Dots define outliers. Letters (a > b) indicate significant difference between HAT and malaria co-infection compared to mono-infection (Kruskal–Wallis, Dunn’s multiple comparisons test, P < 0.05)

The co-infected cases expressed a significantly higher plasma level of IFN-γ, IL-6, and IL-10 (37.78, 127.5, and 205.3 pg/ml) than malaria (P = 0.0132, P < 0.0001, and P < 0.0001), but no significant difference with HAT (P > 0.9999, P = 0.8151, and P = 0.4213) (Fig. 4a, c, d) respectively. The plasma TNF-α level was significantly elevated in co-infection (52.72 pg/ml) over HAT or malaria mono-infection (P = 0.0438 or P = 0.0134) respectively (Fig. 4b). However, plasma TGF-β level was not significantly different between co-infection (1922 pg/ml) and mono-infection of either HAT or malaria (P = 0.7494 or P = 0.2058) respectively (Fig. 4e) (Kruskal–Wallis, Dunn’s multiple comparisons test, P < 0.05).

### Correlation between cytokine levels in HAT and malaria co-infection

**Fig. 5:**
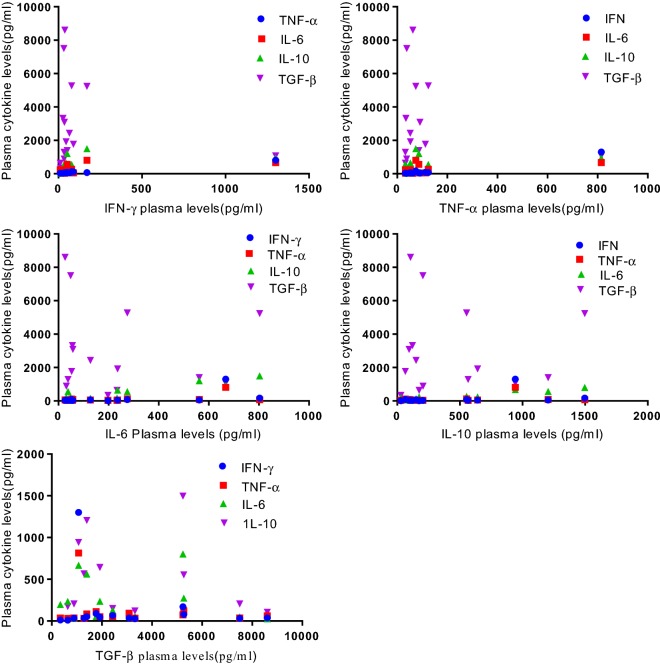
Scatter plots showing correlations between plasma cytokine levels among co-infected cases (n = 15) (Spearman correlation significant, P < 0.05)

Spearman’s correlation rank test was performed to investigate the association between the cytokine levels among the co-infection. Significant positive correlations were observed between IFN-γ with TNF-α (Spearman r = 0.847) and IL-6 with IL-10 (Spearman r = 0.557) among co-infection. There was no significant correlation observed between the other cytokines amongst co-infection (P > 0.05) (Spearman’s rank correlations P < 0.05, 2 tailed; Table 2; Fig. 5).

**Table 2:** Correlation coefficient between cytokine levels among HAT and malaria co-infection

Cytokine	IFN-γ	Cytokine correlation coefficient r_s_			
		TNF-α	IL-6	IL-10	TGF-β
IFN-γ		0.847***	0.471	0.439	0.257
TNF-α			0.431	0.193	0.312
IL-6				0.557*	− 0.135
IL-10					0.014

Spearman’s (r_s_) rank correlations were computed at statistical significance P < 0.05* and P < 0.001***, negative (−) = denotes negative correlation

## Discussion

Malaria has been reported to be prevalent among HAT cases [2, 3, 34, 35]. The prevalence of *P. falciparum* malaria among *T. b. rhodesiense* HAT cases in this study was 46.8% (15/32), which is higher than 28.9% (70/242) earlier reported in Uganda [2]. In the same year, Kato et al. [9] reported 11% (6/55) malaria prevalence among HAT infected cases lower than the current findings, which were excluded from immunological studies as focused on only cytokine response among HAT cases. Studies conducted in the other countries reported 30% HAT and malaria co-infection prevalence in Southern Sudan lower than that of this study [35]. In Kenya, 100% prevalence of HAT and malaria co-infection [3] and 79.7% in Tanzania [1], were notably higher than results of this study. The observed prevalence of co-infection in this study might have been higher, but the reported malaria self-pre-treatment, treatment of malaria before laboratory diagnosis from clinics, Ugandan government programme of free mosquito bed nets supply to every family in the region, spraying of mosquitoes and tsetse flies through the Ministry of Health and other control methods may have minimised the plasmodium and trypanosome infections [27].

Cases with HAT and/or malaria parasites responded with an overall increased secretion of plasma cytokines than healthy controls. Therefore *T. b. rhodesiense* and/or *P. falciparum* infections are associated with up-regulated cytokine responses. A significantly higher level of IFN-γ, TNF-α, IL-6, and IL-10 were expressed by co-infected cases than malaria cases, except for TGF-β which did not vary significantly. Co-existence of HAT and malaria parasites in cases intensely changed their immunological cytokine response relative to malaria. Therefore, *T. b. rhodesiense* co-infection led to a synergistic cytokine response relative to *P. falciparum* infection in host. No significant difference was expressed between co-infected and HAT cases for cytokine IFN-γ, IL-6, IL-10 and TGF-β levels, apart from TNF-α levels which were significantly higher in co-infected than mono-infected HAT cases. This indicates that *P. falciparum* infection modified the TNF-α level synergistically in *T. b. rhodesiense* infected host. The HAT cases expressed significantly higher plasma IFN-γ, IL-6 and IL-10 levels than malaria cases in this study. These findings suggest that *T. b. rhodesiense* significantly induces the host immune cytokine network more than *P. falciparum* infection. No significant difference between HAT and malaria mono-infected cases was noted for TNF-α and TGF-β levels. However, no study explores the cytokine profile concentration of *T. b. rhodesiense* relative to *P. falciparum* in naturally infected humans.

Cases with HAT and/or malaria parasites responded with an overall increase in secretion of pro-inflammatory cytokines (IFN-γ and TNF-α), which partially suggested the effect induced by either HAT or malaria mono-infections. This is in agreement with previous studies described in separate cases of *T. b. rhodesiense* HAT [7–9] or *P. falciparum* malaria [10, 36]. But Da Costa et al. [37], reported that TNF-α level was lower in *P. vivax* malaria than healthy individuals. For trypanosome infection, it has been described that IFN-γ is associated with the penetration of the blood brain barrier in animal models [38]. The TNF-α plays a key role in parasite control and infection pathology in *T. brucei* infections [39, 40], similar to IFN-γ [13, 41]. The magnitude of TNF-α and IFN-γ was associated with the progression and severity of HAT [7, 8]. Previous study by Maclean et al. [7] found higher level of plasma TNF-α in HAT cases from Uganda which was associated with rapid disease progression with increased disease severity compared to HAT cases from Malawi. In another HAT study however, TNF-α of plasma levels was low in cases and not significantly different from the controls [42]. In eastern Uganda, Tororo cases rapidly progressed to the late meningoencephalitic stage with increased disease severity associated with higher levels of plasma IFN-γ relative to Soroti cases despite their close geographical HAT foci [8]. The elevated pro-inflammatory responses observed in plasmodium infections have been described also to protect against pre-erythrocytic stage [43–45], blood stage [43], exo-erythrocytic hepatic stage development [46, 47], resolution of parasitemia [12], and acute clinical malaria manifestation [11, 48]. Secretion of IFN-γ and TNF-α in co-infected cases exhibited a significant positive correlation with each other and was significantly elevated over malaria levels. The differences in TNF-α levels allowed to distinguish the co-infected from both HAT or malaria mono-infected cases, indicating that both diseases synergistically contributed to its elevation. Similar observations have been reported in animal model for IFN-γ and TNF-α between co- and mono-infection of trypanosome and plasmodium. [19]. The current results suggest the presence of elevated plasma IFN-γ level of *T. b. rhodesiense* infection as compared to the *P. falciparum* malaria cases. The main pro-inflammatory IFN-γ response induced by *T. b. rhodesiense* from natural killer (NK) and T cells may partially act as a pre-regulating activation of macrophages against plasmodium infection. This establishes protective adaptive immunity through TNF-α and nitric oxide impairment of hepatic stage of plasmodium [46, 49].

The elevation of pro-inflammatory cytokine response is regulated by anti-inflammatory cytokines. Cases with HAT and/or malaria parasites responded with an overall increased secretion of anti-inflammatory cytokines (IL-6, IL-10 and TGF-β). Similarly, IL-6 and IL-10 increased levels agrees with other studies in HAT [9, 42] or malaria infections [36, 37]. Contrary to this study, IL-10 plasma level was significantly lower in *T. b. gambiense* HAT and *P. falciparum* co-infection than healthy controls. Consequently, IL-10 plasma level of HAT was not significantly affected by *P. falciparum* infection [20], in agreement with the current study. In this study, a positive correlation between IL-6 and IL-10 elevated levels during co-infection was observed. These elevated anti-inflammatory cytokines may account for the low levels of the pro-inflammatory TNF-α and IFN-γ among co-infected cases. This may suggest a regulatory response during co-infection. Therefore, trypanosome and plasmodium parasites have the ability to by-pass the immune system and interact to induce immunosuppression for their co-existence, thus enhancing their co-infection in the host [5]. Also known for its anti-inflammatory role in autoimmune conditions [17], IL-10 also possesses host protective roles against enhanced inflammation in *P. falciparum* malaria [50] and *T. brucei* infection [13, 41]. The role of IL-10 is to control excessive inflammation produced by the pro-inflammatory cytokines during plasmodium infection [18]. Both IL-6 and IL-10 high levels have been described to be responsible for the neuropathy decline in *T. brucei* [41]. Kato et al. [9] showed that plasma IL-6 was negatively associated with splenomegaly in HAT. In the current study, IL-6 and IL-10 levels were significantly elevated in HAT similar to co-infected cases over malaria mono-infection. This suggests that *T. b. rhodesiense* induces these anti-inflammatory cytokines that may regulate the pro-inflammatory cytokine response to *P. falciparum* during co-infection in host. The elevated TGF-β level in HAT over healthy controls was in agreement with a study by Kato et al. [9]. At higher plasma concentrations, TGF-β was associated with protective role in HAT cases from Malawi with slow progress to the late stage compared to Ugandan HAT cases [7]. For malaria, elevated plasma TGF-β levels has been reported in other studies [51, 52] as observed in this study. During early malaria infection in mice, TGF-β release is associated with slow parasite growth hence induction of protective immune response and in late infection down regulates pro-inflammatory immune responses [53]. The suppression of IFN-γ and NO production by TGF-β induces failure of resistance to blood stage malaria [54]. This suggests that parasite growth is associated with elevation of TGF-β in human malaria infections. Therefore, TGF-β modulates the Th1/Th2 immune balance through inhibition or down-regulation of pro-inflammatory responses [16].

The sample size was small and may not be adequate to identify the effect of *T. b. rhodesiense* and/or *P. falciparum* infections on immune cytokine response of the host. This also limited the investigation of the cytokine levels from HAT and/or malaria infections matched by sex and age. Some pathogens for other infections might have been missed as all participants were subjected only to the routine laboratory diagnosis at the hospital associated with low sensitivity. This may explain the presence of outliers in the cytokine levels. Many HAT cases report to the hospital having been medicated or self-medicated on suspicion of malaria when it is actually HAT, since clinical symptoms are quite similar even to other disease conditions. Nevertheless, the study provides information on the immune cytokine response of *T. b. rhodesiense* and/or *P. falciparum* infections. Follow up studies on plasma and CSF cytokine profiles of HAT and malaria co-infections which are stratified would be appropriate. For example, cases with mild, severe, and cerebral malaria or symptomatic and asymptomatic malaria with either early or late stage HAT. This will also harmonise existing controversies about cytokines as potential disease biomarkers. Further studies about antibodies, chemokine and other biomarkers would be very important.

## Conclusion

The prevalence of malaria among HAT cases was high (46.8%). The HAT and/or malaria infection is associated with up-regulated cytokine responses. The *T. b. rhodesiense* infection significantly induced host immune cytokine response of IFN-γ, IL-6, and IL-10 more than *P. falciparum* infection. Malaria and HAT co-infection modified synergistically the pro-inflammatory (IFN-γ, TNF-α) and anti-inflammatory (IL-6, and IL-10) cytokine response than *P. falciparum* mono-infections. The TNF-α plasma level partially indicated the effect induced by mono-infections of HAT and malaria or from a synergistic effect of the co-infection which indicates a protective immunity against the parasites. The up-regulated cytokines may have adverse effects on pathogenesis, prognosis and resolution of the infections.

## Data Availability

The datasets used and/or analysed during the current study are available from the corresponding author on request.

## Abbreviations

ACT: artemisinin-based combination therapy
bp: base pairs
CNS: central nervous system
CSF: cerebrospinal fluid
DNA: deoxyribonucleic acids
ELISA: enzyme linked immuno sorbent assay
HAT: Human African Trypanosomiasis
HIV: human immunodeficiency virus
IFN-γ: interferon-gamma
IL: interleukin
MSP: merozoite surface protein
NO: nitric oxide
OD: optical density
PCR: polymerase chain reaction
PCV: packed cell volume
SRA: serum resistance antigen
TGF-β: transformation growth factor beta
TNF-α: tumor necrosis factor alpha
*T. b. r*: *Trypanosoma brucei rhodesiense*
*T. b. g*: *Trypanosoma brucei gambiense*
UTI: urinary tract infections
WBC: white blood cell
WHO: World Health Organization
